# Annual risk of long-term sickness absence due to musculoskeletal disorders across the lifespan and the role of physical activity and insomnia symptoms: the HUNT Study

**DOI:** 10.1186/s12889-025-22519-4

**Published:** 2025-04-08

**Authors:** Karoline Moe, Eivind Schjelderup Skarpsno, Tom Ivar Lund Nilsen, Paul Jarle Mork, Paulo Ferreira, Lene Aasdahl

**Affiliations:** 1 Department of Public Health and Nursing, Faculty of Medicine and Health Sciences, Norwegian University of Science and Technology, Trondheim, Norway; 2 Department of Neurology and Clinical Neurophysiology, St. Olavs Hospital, Trondheim, Norway; 3 Clinic of Emergency Medicine and Prehospital Care, St. Olav’s Hospital, Trondheim University Hospital, Trondheim, Norway; 4 Musculoskeletal Research Group, School of Health Sciences, Faculty of Medicine and Health, The University of Sydney, Sydney, Australia; 5 Unicare Helsefort Rehabilitation Centre, Rissa, Norway

**Keywords:** Sickness absence, Musculoskeletal disorders, Physical activity, Insomnia symptoms, Chronic musculoskeletal pain

## Abstract

**Background:**

The risk of long-term sickness absence due to musculoskeletal (MSK) disorders could be driven by sleep problems and physical inactivity. However, it is not well explored if these associations differ across the lifespan. The aim of this study was to describe the annual risk of long-term sickness absence due to MSK disorders throughout working life, according to insomnia symptoms and physical activity, in people with and without MSK pain.

**Methods:**

A total of 38,253 working-age individuals (20–62 years) with information on self-reported chronic MSK pain, physical activity, and insomnia symptoms in the third (2006-08) and/or fourth (2017-19) survey of the Norwegian HUNT Study were included. Annual sickness absence up to 5 years after participation was obtained from national registry data. Annual risk of long-term sickness absence due to MSK disorders were estimated as the proportion receiving medical benefits for ≥ 31 consecutive days each calendar year using a Poisson regression model. The model was fitted via generalized estimating equations to account for dependencies in observations and presented according to categories of chronic MSK pain combined with insomnia symptoms (yes, no) or physical activity level (inactive/low, moderate, high).

**Results:**

The average annual proportion with long-term sickness absence due to MSK disorders increased from 3.7% in women < 30 years to 11.3% in women ≥ 50 years, and from 2.7 to 7.1% among men in the same age groups. Annual risk of long-term sickness absence due to MSK disorders was greater among those reporting chronic MSK pain and who also suffered from insomnia symptoms compared to those without any of these conditions. This was particularly evident in age 30–39 and 40–49 years, where the co-occurrence of chronic MSK pain and insomnia symptoms was associated with a 4-fold increased risk of long-term sickness absence due to MSK disorders in women, and an almost 5-fold increased risk in men. The risk of sickness absence did not differ according to physical activity levels.

**Conclusion:**

The annual risk of long-term sickness absence due to MSK disorders in working-age individuals was related to insomnia symptoms, especially among those aged 40 to 60 years, but not to physical activity.

**Supplementary Information:**

The online version contains supplementary material available at 10.1186/s12889-025-22519-4.

## Introduction

Increased life expectancy and lower birth rates challenge the Norwegian welfare state [1], underscoring the importance of keeping people in the workforce as long as possible [2]. Similar to other Scandinavian countries, Norway has a high level of sickness absence, with about 1.2% of the gross domestic product spent on sickness and disability benefits due to musculoskeletal (MSK) disorders (e.g., low back pain, osteoarthritis, fibromyalgia) [3]. Previous studies that have investigated trends in sick leave over the working lifespan lack information on modifiable factors that may influence the sick leave at different stages of life [4, 5]. Studies that have included information on physical health, socioeconomic factors, or family factors have been restricted to sick leave in middle-age [6, 7]. As MSK disorders are the main cause of long-term sickness absence in Norway and other European countries [8], it is important to identify factors that are associated to this type of sickness absence at different stages of life.

Physical inactivity [9–15] and insomnia symptoms [16–20] are both related to increased risk of long-term sickness absence due to MSK disorders, but few studies have examined their influence on sick leave in coexistence with pain status [21] or if this differs across the lifespan. Most adults with chronic MSK pain (commonly defined as pain persisting more than 3 months) do not receive long-term sickness absence despite the high prevalence of chronic MSK pain (8–65%) in the general population [22–24]. However, the risk of long-term sickness absence due to a musculoskeletal disorder could be driven by insomnia symptoms and physical inactivity, and differ by chronic MSK pain status. The evidence suggests that the association between chronic MSK pain and insomnia symptoms is bidirectional [25, 26], but it is likely that people with both conditions have a particularly increased risk of sickness absence due to MSK disorders [21]. Furthermore, physical activity could potentially lower the risk of sick leave due to improved pain severity and physical function, although the evidence is conflicting [24]. Insomnia symptoms and physical activity are influenced by other factors that could act differently at different stages of life such as stress, transitions into the labor market, family dynamics, and health status [27–30]. However, it is not well explored whether insomnia symptoms and physical activity influence the risk of sickness absence differently across the lifespan.

The aim of this study was therefore to describe the annual risk of long-term sickness absence due to MSK disorders throughout working life, and to explore whether this risk is related to insomnia symptoms and physical activity in people with and without chronic MSK pain.

## Methods

### Study sample

This study uses data from the third (2006-08) and fourth (2017-19) survey of the population-based Trøndelag Health Study (HUNT). All inhabitants aged 20 years and above in Nord-Trøndelag County, Norway, were invited to participate. Details of these surveys are described elsewhere [31–33]. Registry data from the Norwegian Labour and Welfare Administration (NAV) were used to obtain data on medical benefits, including dates and diagnoses. Registry data comprising information about educational attainment were obtained from Statistics Norway. The unique personal identification number held by all Norwegian citizens was used to link data from the different sources.

**Fig. 1:**
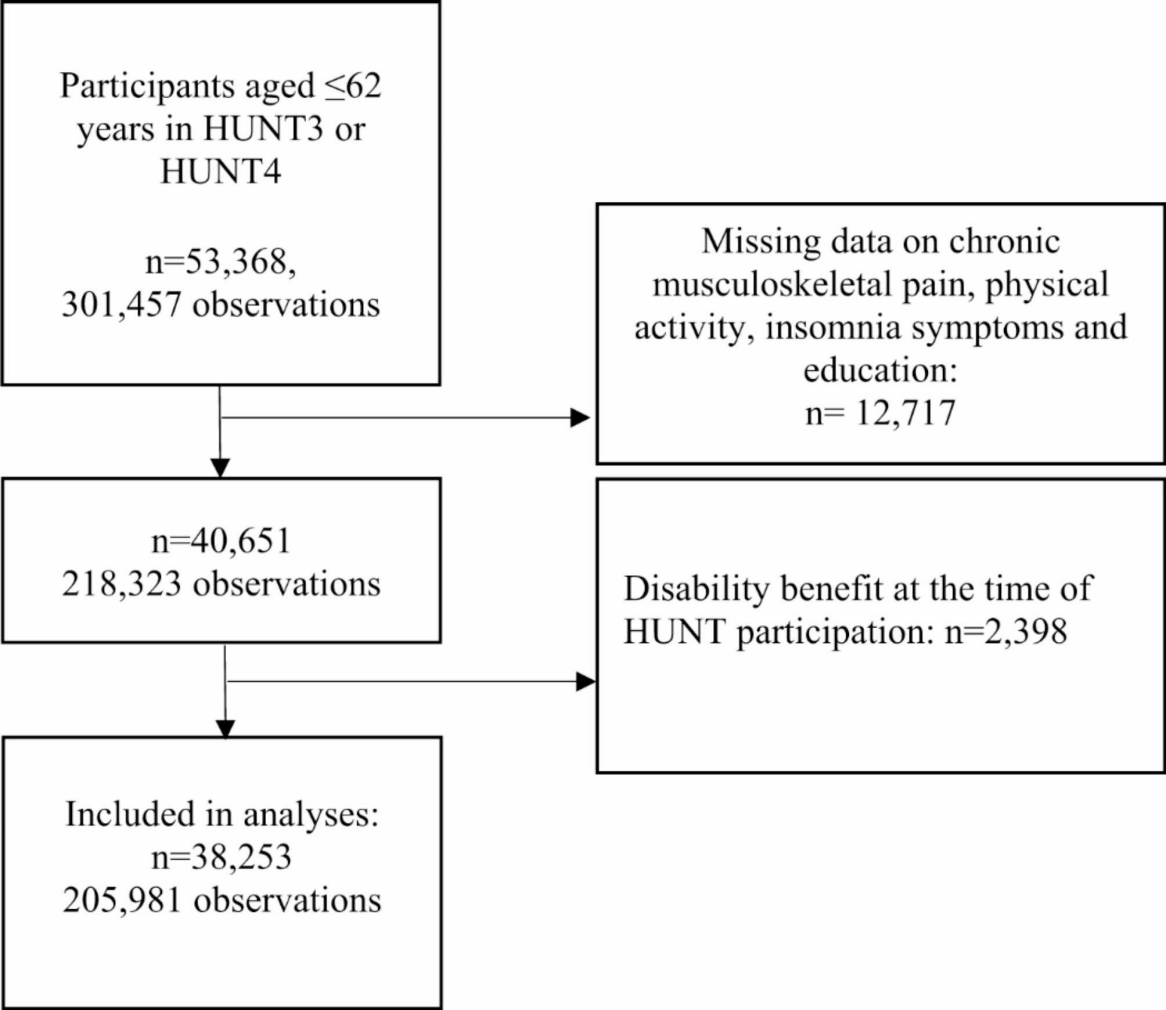
Flowchart for the selection of the study population

A total of 53,368 participants aged 20–62 years participated in at least one of the HUNT surveys. Participants were excluded if they lacked data on study variables. Of those 12,717 excluded, 94% had missing data on chronic MSK pain, 37% on insomnia symptoms, 4% on physical activity, and 2% on education (Fig. 1). A total of 2,398 participants were excluded as they received disability benefits (all causes) prior to or the year of participation in the respective HUNT survey. Therefore, the current study included 38,253 participants (16,519 men and 21,734 women), contributing with a total of 205,981 observations to the study.

### Long-term sickness absence

In Norway, the employer pays the first 16 days of a sick leave period, and then NAV constitute 100% of the wage loss for medically certified sick leave up to 12 months. In situations where the employees has not returned to work after 12 months, they may apply for more long-term medical benefits, such as work assessment allowance and disability benefits. In contrast to sick leave, these benefits cover about 66% of the wage loss. Work assessment allowance can last for up to four years (could be prolonged in some cases), during which attendance to work training or work specific rehabilitation is mandatory unless prevented by medical reasons. If work rehabilitation is insufficient or inappropriate, the employee may apply for disability benefits when the earning capacity is permanently reduced by at least 50% due to illness or injury.

Sickness absence was defined as any type of medical benefits (sick leave, work assessment allowance or equivalent) except permanent disability benefits. Medically certified sickness absence can be graded from 20 to 100%, independent of the employment fraction. Long-term sickness absence was defined as being partly or fully absent from work due to sickness absence that lasted at least 31 consecutive days. Before 2010, there was unreliable information on diagnoses and time periods for two out of three types of medical benefits that constitutes work assessment allowance. These events are therefore excluded from the risk estimation. However, based on available data, it is likely that these cases constitute less than 1% of all medical benefits during this period. Medical benefits due to MSK disorders were identified based on the 9th and 10th revisions of the International Classification of Diseases (ICD-10 codes M00-M99 and their ICD-9 equivalents) and the International Classification of Primary Care (ICPC1 and ICPC2, codes L00-L99). The relevant diagnoses for each sickness absence were set by the physician who certified the sickness absence.

### Physical activity, insomnia symptoms, and chronic musculoskeletal pain

Chronic MSK pain was assessed by a question from the Standardised Nordic questionnaires for the analysis of musculoskeletal symptoms [34]; “In the last year, have you had pain or stiffness in muscles or joints that has lasted at least 3 consecutive months?”, with response options “yes” and “no”.

Leisure time physical activity per week was assessed by three questions previously validated [35]; 1) “How often do you exercise?” (“never”, “less than once a week”, “once a week”, 2–3 times a week”, “nearly every day”), 2) “How hard do you exercise? (average) (If you exercise once or several times a week: )” (“I take it easy, I don’t get out of breath or break a sweat”, “I push myself until I’m out of breath or break into a sweat”, “I practically exhaust myself”), and 3) “How long do you exercise each time? (average) (If you exercise once or several times a week: )” (“less than 15 minutes”, “15–29 minutes”, “30–60 minutes”, “more than one hour”). Based on the information containing frequency, intensity, and duration, participants were classified into three categories: (1) “inactive/low activity” (< 3 h light and no hard activity), (2) “moderate activity” (at least ≥ 3 h light and/or < 1 h hard activity), and (3) “high activity” (any light and ≥ 1 h hard activity).

Insomnia symptoms were assessed by the questions; “How often in the last 3 months have you:” 1) “Woken too early and could not get back to sleep”, 2) “Had difficulty falling asleep at night”, 3) “Woken up repeatedly during the night”, with the alternatives “never/seldom”, “sometimes”, and “several times a week” (“at least three times a week” in HUNT4) [36]. Participants were categorized as having insomnia symptoms if they had answered “several times a week”/”at least three times a week” on at least one of the three questions.

### Other variables

Information on completed education at the same year as participation in the respective HUNT survey was obtained from Statistics Norway [37], with the categories (1) “unknown or no completed education”, (2) “compulsory school level”, (3) “upper secondary school level”, (4) “tertiary vocational education level”, (5) “higher education- undergraduate level”, and (6) “higher education- postgraduate level”.

### Statistical analysis

Information from the HUNT surveys were applied from 1st of January the year of participation and until (1) 5 years after participation, (2) death or emigration, (3) all-cause disability benefits, or (4) 31st of December 2021. Data on medical benefits were measured annually up to 5 years after participation in the respective HUNT surveys or until 31st of December 2021. Participants’ characteristics were summarized within 10-year age categories (< 30, 30–39, 40–49, ≥ 50 years) separately for men and women.

The sex-specific annual risk of long-term sickness absence across the working life was estimated as the proportion receiving long-term (≥ 31 days) medical benefits due to MSK disorders each of the five calendar years from participation in a HUNT survey using a Poisson regression model via a generalized estimating equation model to account for dependencies in observations (i.e., participation in both surveys, repeated measures of sickness absence) and controlling for education and HUNT survey. The predicted annual proportions of long-term sickness absence were calculated for each combination of age (year), chronic MSK pain status (no, yes), and the physical activity or insomnia symptoms categories and presented as margins plots with 95% confidence intervals (CIs). In similar models, we also estimated the average annual proportions within 10-year age categories, with corresponding absolute and relative differences in risk with 95% CIs.

### Sensitivity analyses

Possible effect modification was assessed by the estimated the relative excess risk due to interaction (RERI), which measure interaction on an additive scale [38]. A RERI value > 0 indicates a synergistic effect beyond additivity. To further explore the possible role of insomnia severity we also analyzed insomnia classified as number of symptoms (“0”, “1”, “≥2” symptoms). As the status of physical activity, insomnia symptoms, and chronic MSK pain may change over time, we repeated the analyses only using data on sickness absence for the first two years after participation in the respective HUNT survey. To illustrate the total absence from work, including permanent medical benefits, we also repeated the main analysis additionally including permanent disability benefits due to MSK disorders as the outcome. Participants that received disability benefits due to other diagnoses were excluded at the time point they received the benefit (competing risk). Finally, to assess the total amount of absence from work, irrespective of diagnosis, we also conducted similar analyses using sickness absence due to all-cause as the outcome. All analyses were performed using Stata statistical software (version 18) [39].

### Ethics

The study was approved by the Regional Committee for Medical and Health Research Ethics in Central Norway (Ref. no. 230429). The results are presented according to the STROBE statement [40].

**Table 1:** Descriptive information of the study sample, stratified by sex and age group

	Women					Men			
	< 30 years	30–39 years	40–49 years	≥ 50 years		< 30 years	30–39 years	40–49 years	≥ 50 years
No. of observations^a^	15,452	23,538	34,396	46,341		8,463	14,348	24,311	39,132
Annual SA^b^, mean days (SD)	161 (121)	183 (120)	193 (122)	192 (120)		150 (112)	161 (113)	162 (116)	171 (116)
Annual SA^b^, median days (IQR)	111 (214)	148 (232)	161 (253)	162 (237)		108 (175)	121 (181)	120 (208)	135 (206)
Annual SA ≥ 31 days, %	3.7	6.7	9.4	11.3		2.7	4.6	5.7	7.1
Chronic MSK pain^c^, %									
Yes	34.6	42.6	52.5	59.4		28.3	34.8	57.7	49.0
No	65.4	57.4	47.5	40.6		71.7	65.2	42.3	51.0
Physical activity^d^, %									
Inactive/low	31.5	35.6	32.6	34.9		32.6	40.0	41.3	39.8
Moderate	14.1	16.6	15.1	14.1		11.7	14.5	15.0	15.4
High	54.4	47.8	52.3	51.0		55.7	45.5	43.7	44.8
Insomnia symptoms^e^, %									
Yes	27.1	24.5	25.0	34.4		16.5	17.1	17.5	21.9
No	72.9	75.5	75.0	65.6		83.5	82.9	82.5	78.1

Abbreviations: IQR = interquartile range, MSK = musculoskeletal, SA = sickness absence due to musculoskeletal disorders, SD = standard deviation

^a^ Number of observations is the total number of years the participants contributed to the analysis

^b^ Among those with long-term (≥ 31 days) sickness absence due to MSK disorders

^c^ Reported chronic MSK pain lasting for at least 3 months during the past 12 months

^d^ “inactive/low” (< 3 h light and no hard activity), “moderate” (at least ≥ 3 h light and/or < 1 h hard activity), and “high” (any light and ≥ 1 h hard activity)

^e^ Reported at least one insomnia symptom several times a week/at least three times a week

## Results

The annual mean number of sickness absence days due to MSK disorders increased from 6 (SD 38) days in the age group < 30 years to 22 (SD 73) days in the age group ≥ 50 years among women, whilst the corresponding numbers for men were 4 (SD 31) days and 13 (SD 54) days, respectively (Table 1). The average annual proportion of long-term sickness absence due to MSK disorders increased with age for both women (from 3.7% in those < 30 years to 11.3% in those ≥ 50 years) and men (from 2.7 to 7.1%) (Table 1). Among those reporting chronic MSK pain, 6.9% in the youngest age category and 15.8% in the oldest age category among women experienced long-term sickness absence due to MSK disorders, while the corresponding numbers for men were 4.6% and 11.1%.

Participants who were excluded due to missing data had a similar male-to-female ratio (42% vs. 43%), no large difference in mean age (42.8 (standard deviation (SD) 11.7) vs. 45.7 (SD 11.0)), and largely similar proportions with chronic MSK pain (50.6% vs. 47.5%), insomnia symptoms (27.6% vs. 24.9%), low physical activity (42.1% vs. 36.3%), and annual long-term sickness absence due to MSK (7.6% vs. 7.2%). However, the proportion with education below university level was slightly higher than in the analytic sample (67.2% vs. 59.9%).

### Long-term sickness absence related to chronic MSK pain, physical activity, and insomnia symptoms

**Fig. 2:**
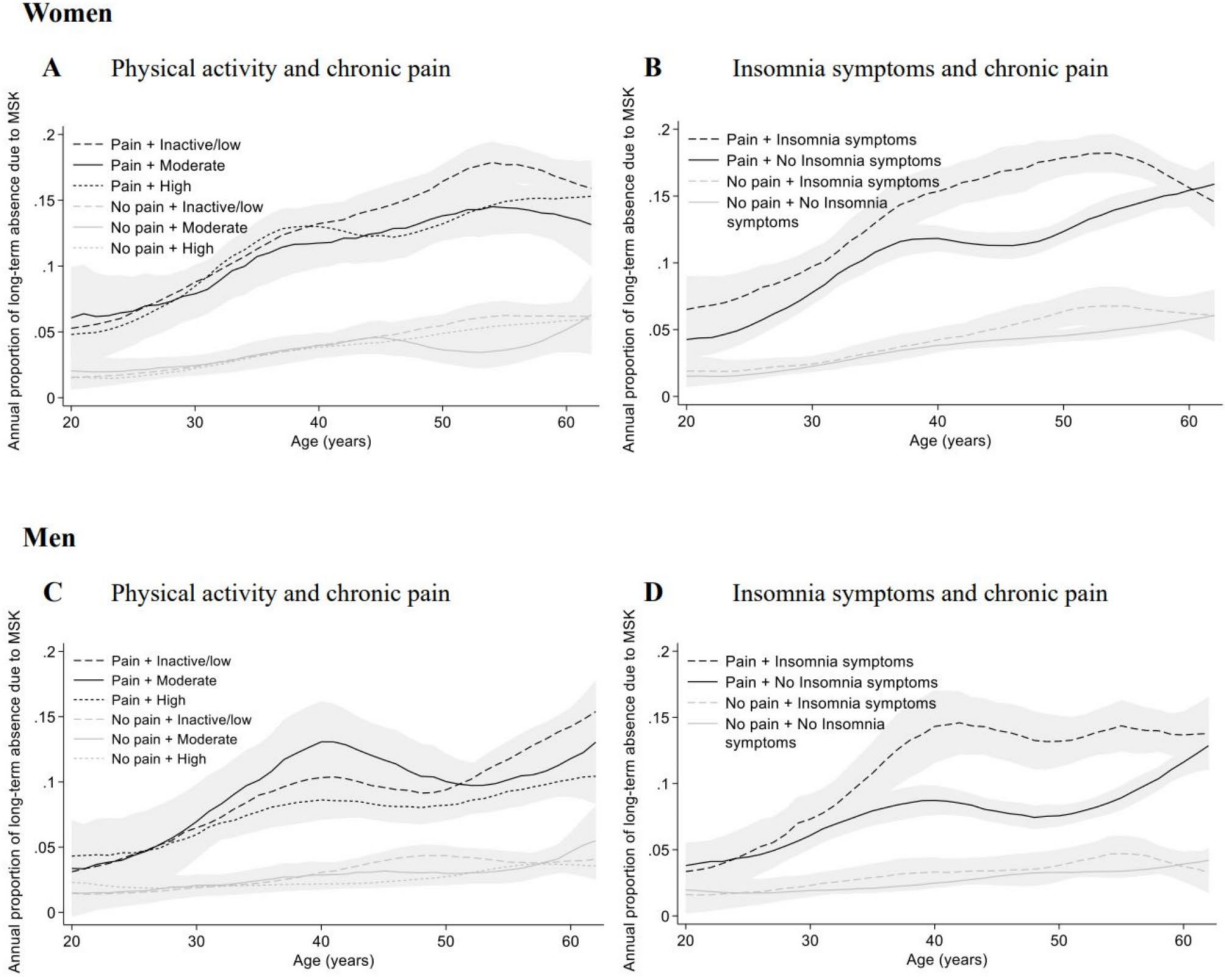
**A-D.** Annual risk of long-term (≥ 31 days) sickness absence due to musculoskeletal (MSK) disorders according to chronic MSK pain, and physical activity or insomnia symptoms

Combining chronic MSK pain status with either physical activity levels or insomnia symptoms showed substantial differences in the annual risk of sickness absence between individuals with and without chronic MSK pain. Only minor differences were observed between the physical activity categories (Fig. 2A-D and Table S1). For insomnia symptoms there was however substantial differences within those reporting chronic MSK pain combined with the presence or absence of insomnia symptoms, mainly between the ages 40 to 60 years for both sexes (Fig. 2A-D). Overall, the average annual risk of long-term sick leave due to MSK disorders increased steadily for each 10-year age category (Table S1).

Absolute risk differences (RD) and relative risk (RR) using those without chronic MSK pain and either no insomnia symptoms or high physical activity as reference are presented in Table S1. Differences in risk among those with chronic MSK pain was observed between those with and without insomnia symptoms in middle aged workers (Fig. 2). In the age categories 30–39 and 40–49, the average annual risk of long-term sickness absence due to MSK disorders increased 4-fold among women and almost 5-fold among men (Table S1).

### Sensitivity analysis

The estimated RERI suggest no substantial synergistic effects between physical activity and chronic MSK pain for those aged ≥ 50 years (RERI 0.74), nor between insomnia symptoms and chronic MSK pain for men ≥ 40 years (RERI 1.82) or women between 30 and 50 years (RERI 1.02) (Table S2). The analyses with three categories of insomnia symptoms showed a stepwise increase in the annual risk of long-term sickness absence with increasing number of insomnia symptoms, among those aged 40 to 60 years who reported chronic MSK pain (Table S3). Restricting the follow-up to two years after the respective HUNT surveys, showed an overall but not statistically significant attenuation in predicted annual risk of sickness absence in the groups not reporting chronic MSK pain, irrespective of physical activity level or insomnia symptoms (Table S4). The reduction in annual risks had some impact on the corresponding RRs, where the risk was slightly increased from the main analyses in those reporting chronic MSK pain. When disability benefits due to MSK disorders was included in the outcome of long-term sickness absence due to MSK disorders, the annual risk of long-term sick leave remained similar to the main analysis, except for a slight increase among those ≥ 50 years reporting pain. This increase in annual risk was irrespective of physical activity level or insomnia symptoms (up to 8.0% points increase for women and 5.9% points increase for men) (Table S5).

In analysis of sickness absence due to all causes, the clearest distinction of annual risks was between chronic MSK pain status, similar to sickness absence due to MSK disorders (Table S6). The trend deviated somewhat from the main analysis in women when examining chronic MSK pain in combination with physical activity, as there was no clear increase in annual risk of sickness absence after the age of 40 (Table S6). When examining chronic MSK pain and insomnia symptoms, the trend was somewhat different compared to sickness absence due to MSK disorders, as there was no difference in annual risk between “pain and no insomnia symptoms” and “no pain and insomnia symptoms” in men and these groups only differed in the age span 40–60 years in women (Table S6).

## Discussion

This population-based study supports that the average annual risk of long-term sickness absence due to MSK disorders steadily increase with age. Moreover, the results indicate that individuals with chronic MSK pain who also reported insomnia symptoms had a higher annual risk of sickness absence, particularly between the ages 40 to 60 years. Minor differences in sickness absence were observed between the different levels of physical activity, regardless of chronic MSK pain status.

Our findings are in line with existing knowledge about sex differences and age-related trends for sickness absence due to MSK disorders [41–43]. However, the current study expands the knowledge by providing a more detailed insight into how these trends are related to insomnia symptoms or engagement in physical activity in individuals with or without chronic MSK pain. While MSK disorders are well-documented as a leading cause of sickness absence [44–46], this study shows that individuals suffering from both insomnia symptoms and chronic MSK pain had a significantly higher probability of long-term sickness absence, particularly in the age group 40–60 years. A possible explanation for this is that those who struggle with insomnia symptoms may have more complex or severe symptoms of MSK pain. This relation could be driven by the link between poor sleep and elevated pain sensitivity, fatigue, and impaired physical and mental functioning [47], which makes them more prone to activity limiting MSK pain and work disability. However, insomnia symptoms seemed to have more influence in those with pain over the age of 40, suggesting that this association is enhanced with increasing age. Our assessment of insomnia symptoms did not differentiate between mild and severe sleep problems. Previous studies have suggested an increased risk of more severe sleep problems (e.g., insomnia and sleep apnea) in middle-age and older individuals, although the findings are conflicting [48, 49]. Thus, it is possible that participants classified with insomnia symptoms in the older age-groups had more severe insomnia symptoms than the younger individuals.

Regular physical activity has been shown to be inversely related to the risk of several chronic conditions [50] and individuals who engage in leisure time physical activity have also a reduced risk of sickness absence [9–15]. However, our findings showed no clear difference in the annual risk of sickness absence due to MSK between those reporting high and low physical activity levels, irrespective of their chronic MSK pain status. Previous studies suggest a bidirectional relationship between physical activity and chronic MSK pain [51, 52], which makes it challenging to explain the association between physical activity and long-term sickness absence due to MSK. As our measure of chronic MSK pain does not assess pain severity, the impact of MSK pain may widely differ within this group. Although we did not observe a clear advantage in favor of a higher activity level on the probability of long-term sickness absence, promoting leisure time physical activity still holds value as a preventive measure, as sedentary behavior is associated with other non-communicable conditions, and mortality [53, 54].

### Strengths and limitations

A major strength of this study is its large population-based sample, which provided comprehensive data on chronic MSK pain, physical activity, and insomnia symptoms linked with detailed registry-based data on sickness absence and education. The longitudinal design also allowed follow up of sickness absence over time, providing valuable insights into the dynamics of sickness absence in relation to age, chronic MSK pain, physical activity, and insomnia symptoms. The study provides data on sickness absence due to MSK disorders as well as all-cause, which gives important insights in cause-specific absence but also in the context of all types of sickness absence. Our study still has some limitations. Firstly, we used self-reported measures of physical activity that could induce information bias [55]. Additionally, we allowed the chronic MSK pain status, physical activity and insomnia symptoms to apply for up to 5 years, which do not take into account fluctuations of symptoms, remission of MSK pain and/or insomnia symptoms, or development of new cases of sickness absence during the specified time period. However, the sensitivity analysis restricting information to apply for two years did not impact our results. Secondly, the threshold for having insomnia symptoms could be considered as low, and do not differentiate between mild and severe insomnia symptoms. However, a sensitivity analysis showed a stepwise increase in risk of long-term sickness absence with increasing number of insomnia symptoms among those reporting chronic MSK pain. Also, chronic MSK pain does not capture differences in severity of symptoms or if the pain is activity limiting, which may have influenced our results. Finally, the results of this study are based on complete case analyses among participants in the HUNT Study. However, the proportion reporting insomnia symptoms or low physical activity were only 2–6% higher in participants who were excluded from the analyses, with almost similar characteristics on sex, age and proportion on long-term sickness absence due to MSK.

## Conclusion

The average annual risk of long-term sickness absence in Norway due to MSK disorders increased steadily by age. We found that individuals reporting chronic MSK pain combined with insomnia symptoms had substantially higher annual risk of sickness absence due to MSK disorders than those without insomnia symptoms, especially between the ages 40 to 60 years. There was no indication that the risk of long-term sickness absence was related to physical activity levels. The results emphasize that addressing insomnia symptoms among middle-aged individuals with chronic MSK pain should be a public health priority, but it is uncertain whether this is an effective measure to reduce long-term sickness absence.

## Electronic supplementary material

Below is the link to the electronic supplementary material.

Supplementary Material 1

## Data Availability

No datasets were generated or analysed during the current study.

## Abbreviations

CI: Confidence interval
HUNT: The Trøndelag Health Study
IQR: Interquartile range
MSK: Musculoskeletal
NAV: The Norwegian labour and welfare administration
RR: Relative risk
RD: Risk difference
SA: Sickness absence due to musculoskeletal disorders
SD: Standard deviation
STROBE: Strengthening the reporting of observational studies in epidemiology
