# Supplementary material for: Annual risk of long-term sickness absence due to musculoskeletal disorders across the lifespan and the role of physical activity and insomnia symptoms: the HUNT Study
Source: BMC Public Health. 2025 Apr 8;25:1318. doi: 10.1186/s12889-025-22519-4 (PMC11978103; doi:10.1186/s12889-025-22519-4)
Supplement: Supplementary file 1 — Supplementary Material 1 [file 12889_2025_22519_MOESM1_ESM.docx]

**Table S1.** Annual proportions (risks), risk differences (RDs) and relative risks (RRs) of long-term sickness absence due to musculoskeletal (MSK) disorders according to chronic MSK pain^a^ in combination with physical activity or insomnia symptoms in different age groups, by sex.

|  | <30 years | | | 30-39 years | | | 40-49 years | | | ≥50 years | | |
| --- | --- | --- | --- | --- | --- | --- | --- | --- | --- | --- | --- | --- |
|  | Risk  (95% CI) | RD  (95% CI) | RR  (95% CI) | Risk  (95% CI) | RD  (95% CI) | RR  (95% CI) | Risk  (95% CI) | RD  (95% CI) | RR  (95% CI) | Risk  (95% CI) | RD  (95% CI) | RR  (95% CI) |
| **Men** |  |  |  |  |  |  |  |  |  |  |  |  |
| Physical activity^b^ |  |  |  |  |  |  |  |  |  |  |  |  |
| No Pain- High | 2.0  (1.4-2.5) | Ref. | Ref. | 2.1  (1.7-2.5) | Ref. | Ref. | 2.4  (2.0-2.8) | Ref. | Ref. | 3.4  (3.0-3.9) | Ref. | Ref. |
| No Pain- Moderate | 1.7  (0.7-2.7) | -0.2  (-1.4-0.9) | 0.88  (0.32-1.43) | 2.5  (1.6-3.4) | 4.9  (-0.6-1.4) | 1.19  (0.70-1.68) | 3.0  (2.2-3.8) | 0.7  (-0.2-1.5) | 1.27  (0.89-1.66) | 3.7  (2.9-4.5) | 0.3  (-0.7-1.2) | 1.08  (0.80-1.35) |
| No Pain- Inactive/low | 1.6  (0.9-2.2) | -0.4  (-1.2-0.4) | 0.80  (0.41-1.19) | 2.4  (1.9-2.9) | 3.0  (-0.4-1.0) | 1.14  (0.80-1.48) | 3.9  (3.3-4.5) | 1.5  (0.8-2.2) | 1.63  (1.27-2.00) | 4.0  (3.4-4.6) | 0.6  (-0.2-1.3) | 1.16  (0.94-1.39) |
| Pain- High | 4.9  (3.4-6.4) | 2.9  (1.3-4.5) | 2.49  (1.47-3.51) | 7.7  (6.3-9.1) | 5.6  (4.2-7.1) | 3.69  (2.69-4.69) | 8.3  (7.2-9.4) | 5.9  (4.7-7.0) | 3.47  (2.75-4.18) | 9.4  (8.5-10.3) | 6.0  (5.0-7.0) | 2.75  (2.31-3.20) |
| Pain- Moderate | 5.5  (2.3-8.7) | 3.5  (0.3-6.8) | 2.80  (1.00-4.61) | 10.6  (8.0-13.3) | 8.5  (5.8-11.2) | 5.08  (3.44-6.72) | 11.4  (9.2-13.6) | 9.0  (6.8-11.3) | 4.79  (3.58-5.99) | 10.8  (9.3-12.3) | 7.4  (5.8-9.0) | 3.15  (2.54-3.75) |
| Pain- Inactive/low | 4.9  (3.4-6.5) | 3.0  (1.3-4.6) | 2.52  (1.46-3.58) | 9.1  (7.5-10.6) | 7.0  (5.4-8.6) | 4.34  (3.19-5.49) | 9.7  (8.5-10.8) | 7.3  (6.1-8.5) | 4.05  (3.24-4.85) | 12.5  (11.5-13.5) | 9.1  (8.0-10.1) | 3.64  (3.08-4.20) |
| Insomnia symptoms^c^ |  |  |  |  |  |  |  |  |  |  |  |  |
| No Pain- No | 1.8  (1.4-2.2) | Ref. | Ref. | 2.2  (1.8-2.5) | Ref. | Ref. | 3.0  (2.6-3.3) | Ref. | Ref. | 3.6  (3.2-3.9) | Ref. | Ref. |
| No Pain- Yes | 1.8  (0.9-2.8) | 0.0  (-1.0-1.1) | 1.02  (0.44-1.60) | 2.9  (1.9-3.9) | 0.8  (-0.3-1.8) | 1.35  (0.85-1.84) | 3.5  (2.5-4.5) | 0.5  (-0.5-1.6) | 1.17  (0.81-1.54) | 4.2  (3.3-5.0) | 0.6  (-0.3-1.6) | 1.17  (0.90-1.44) |
| Pain- No | 4.9  (3.7-6.1) | 3.1  (1.8-4.4) | 2.74  (1.81-3.66) | 7.9  (6.9-9.0) | 5.8  (4.7-6.8) | 3.66  (2.93-4.40) | 7.9  (7.2-8.7) | 5.0  (4.1-5.8) | 2.68  (2.28-3.08) | 9.8  (9.1-10.5) | 6.3  (5.5-7.0) | 2.76  (2.42-3.09) |
| Pain- Yes | 5.2  (3.3-7.2) | 3.4  (1.4-5.4) | 2.91  (1.63-4.20) | 11.2  (9.0-13.5) | 9.1  (6.8-11.4) | 5.19  (3.87-6.52) | 13.9  (12.0-15.7) | 10.9  (9.0-12.8) | 4.67  (3.85-5.50) | 13.9  (12.5-15.2) | 10.3  (8.9-11.6) | 3.89  (3.36-4.42) |
| **Women** |  |  |  |  |  |  |  |  |  |  |  |  |
| Physical activity^b^ |  |  |  |  |  |  |  |  |  |  |  |  |
| No Pain- High | 1.7  (1.3-2.1) | Ref. | Ref. | 3.1  (2.7-3.6) | Ref. | Ref. | 4.3  (3.8-4.8) | Ref. | Ref. | 5.5  (4.9-6.1) | Ref. | Ref. |
| No Pain- Moderate | 2.2  (1.3-3.0) | 0.5  (-0.5-1.4) | 1.27  (0.70-1.84) | 3.3  (2.4-4.2) | 0.1  (-0.9-1.2) | 1.05  (0.72-1.38) | 4.2  (3.3-5.2) | -0.1  (-1.2-1.0) | 0.99  (0.73-1.24) | 4.2  (3.2-5.2) | -1.3  (-2.4- -0.2) | 0.77  (0.57-0.96) |
| No Pain- Inactive/low | 1.9  (1.4-2.4) | 0.2  (-0.5-0.9) | 1.12  (0.72-1.52) | 3.2  (2.6-3.8) | 0.1  (-0.7-0.8) | 1.03  (0.79-1.27) | 4.7  (4.0-5.5) | 0.4  (-0.5-1.4) | 1.10  (0.88-1.32) | 6.1  (5.3-6.9) | 0.6  (-0.3-1.6) | 1.11  (0.93-1.30) |
| Pain- High | 6.3  (5.1-7.4) | 4.6  (3.4-5.8) | 3.69  (2.63-4.75) | 11.8  (10.5-13.0) | 8.6  (7.3-10.0) | 3.75  (3.05-4.45) | 12.6  (11.6-13.6) | 8.3  (7.2-9.4) | 2.93  (2.51-3.52) | 14.7  (13.8-15.6) | 9.2  (8.2-10.3) | 2.68  (2.36-3.00) |
| Pain- Moderate | 6.9  (4.4-9.3) | 5.2  (2.7-7.6) | 4.05  (2.37-5.73) | 10.5  (8.5-12.5) | 7.4  (5.3-9.4) | 3.35  (2.54-4.16) | 12.7  (10.9-14.5) | 8.4  (6.5-10.3) | 2.96  (2.40-3.52) | 14.1  (12.5-15.6) | 8.6  (6.9-10.2) | 2.57  (2.18-2.95) |
| Pain- Inactive/low | 6.9  (5.3-8.5) | 5.2  (3.5-6.9) | 4.06  (2.75-5.37) | 11.5  (10.1-12.9) | 8.4  (6.9-9.9) | 3.67  (2.96-4.38) | 14.6  (13.3-15.9) | 10.3  (8.9-11.7) | 3.41  (2.89-3.92) | 17.1  (16.1-18.2) | 11.6  (10.4-12.9) | 3.12  (2.74-3.50) |
| Insomnia symptoms^c^ |  |  |  |  |  |  |  |  |  |  |  |  |
| No Pain- No | 1.7  (1.4-2.1) | Ref. | Ref. | 3.1  (2.8-3.5) | Ref. | Ref. | 4.2  (3.8-4.7) | Ref. | Ref. | 5.2  (4.7-5.7) | Ref. | Ref. |
| No Pain- Yes | 2.1  (1.4-2.8) | 0.3  (-0.5-1.1) | 1.19  (0.73-1.65) | 3.3  (2.6-4.1) | 0.2  (-0.7-1.1) | 1.07  (0.78-1.35) | 5.2  (4.2-6.2) | 1.0  (-0.01-2.1) | 1.23  (0.97-1.50) | 6.5  (5.5-7.5) | 1.3  (0.2-2.4) | 1.25  (1.03-1.47) |
| Pain- No | 5.8  (4.7-6.8) | 4.0  (3.0-5.1) | 3.30  (2.48-4.13) | 10.7  (9.8-11.7) | 7.6  (6.5-8.6) | 3.42  (2.90-3.94) | 11.6  (10.8-12.4) | 7.3  (6.4-8.3) | 2.74  (2.40-3.07) | 14.3  (13.6-15.1) | 9.1  (8.2-10.0) | 2.76  (2.47-3.05) |
| Pain- Yes | 8.0  (6.3-9.6) | 6.2  (4.5-7.9) | 4.57  (3.30-5.84) | 13.1  (11.4-14.8) | 10.0  (8.2-11.7) | 4.17  (3.43-4.90) | 16.9  (15.4-18.3) | 12.6  (11.1-14.2) | 3.99  (3.46-4.52) | 17.1  (16.1-18.1) | 11.9  (10.8-13.0) | 3.29  (2.93-3.64) |

Abbreviations: CI= confidence interval; RD= risk difference; RR= relative risk; Ref.= reference.
^a^ Reported chronic MSK pain lasting for at least 3 months during the past 12 months.
^b^ “inactive/low” (<3 hours light and no hard activity), “moderate” (at least ≥3 hours light and/or <1 hour hard activity), and “high” (any light and ≥1 hour hard activity).
^c^ Reported at least one insomnia symptom several times a week.

**Table S2.** Relative excess risk due to interaction (RERI), estimated from the adjusted risk ratios of the joint effect of physical activity or insomnia symptoms, and chronic musculoskeletal (MSK) pain.

|  |  |  | <30 years | |  |  | 30-39 years | |  |  | 40-49 years | |  |  | ≥50 years | |
| --- | --- | --- | --- | --- | --- | --- | --- | --- | --- | --- | --- | --- | --- | --- | --- | --- |
|  |  |  | RERI (95% CI) | |  |  | RERI (95% CI) | |  |  | RERI (95% CI) | |  |  | RERI (95% CI) | |
| **Men** |  |  |  | |  |  |  | |  |  |  | |  |  |  | |
| Physical activity and chronic MSK pain |  |  | 0.23 | (-0.93 to 1.39) |  |  | 0.51 | (-0.50 to 1.53) |  | - | 0.06 | (-0.79 to 0.67) |  |  | 0.74 | (0.32 to 1.16) |
| Insomnia symptoms and chronic MSK pain |  |  | 0.16 | (-1.24 to 1.55) |  |  | 1.18 | (-0.05 to 2.41) |  |  | 1.82 | (1.06 to 2.59) |  |  | 0.94 | (0.46 to 1.42) |
|  |  |  |  |  |  |  |  |  |  |  |  |  |  |  |  |  |
| **Women** |  |  |  |  |  |  |  |  |  |  |  |  |  |  |  |  |
| Physical activity and chronic MSK pain |  |  | 0.25 | (-0.98 to 1.49) |  | - | 0.10 | (-0.75 to 0.56) |  |  | 0.36 | (-0.07 to 0.79) |  |  | 0.33 | (0.03 to 0.63) |
| Insomnia symptoms and chronic MSK pain |  |  | 1.08 | (-0.11 to 2.27) |  |  | 0.69 | (0.22 to 1.36) |  |  | 1.02 | (0.56 to 1.48) |  |  | 0.28 | (-0.04 to 0.60) |

Abbreviations: CI= confidence interval; MSK= musculoskeletal; RERI= relative excess risk due to interaction

**Table S3.** Annual proportions (risks), risk differences (RDs) and relative risks (RRs) of long-term sickness absence due to musculoskeletal (MSK) disorders according to chronic MSK pain^a^ in combination with number of insomnia symptoms in different age groups, by sex.

|  | <30 years | | | 30-39 years | | | 40-49 years | | | ≥50 years | | |
| --- | --- | --- | --- | --- | --- | --- | --- | --- | --- | --- | --- | --- |
|  | Risk  (95% CI) | RD  (95% CI) | RR  (95% CI) | Risk  (95% CI) | RD  (95% CI) | RR  (95% CI) | Risk  (95% CI) | RD  (95% CI) | RR  (95% CI) | Risk  (95% CI) | RD  (95% CI) | RR  (95% CI) |
| **Men** |  |  |  |  |  |  |  |  |  |  |  |  |
| Insomnia symptoms^b^ |  |  |  |  |  |  |  |  |  |  |  |  |
| No Pain- 0 | 1.8  (1.4-2.2) | Ref. | Ref. | 2.2  (1.8-2.5) | Ref. | Ref. | 3.0  (2.6-3.3) | Ref. | Ref. | 3.6  (3.2-3.9) | Ref. | Ref. |
| No Pain- 1 | 1.6  (0.6-2.6) | -0.2  (-1.3-0.9) | 0.90  (0.30-1.49) | 2.9  (1.8-4.1) | 0.8  (-0.4-2.0) | 1.35  (0.78-1.92) | 3.9  (2.6-5.1) | 0.9  (-0.4-2.2) | 1.31  (0.87-1.76) | 4.1  (3.1-5.1) | 0.5  (-0.6-1.6) | 1.14  (0.84-1.45) |
| No Pain- ≥2 | 3.2  (0.4-6.1) | 1.4  (-1.4-4.3) | 1.80  (0.17-3.43) | 2.7  (1.1-4.4) | 0.6  (-1.1-2.3) | 1.27  (0.48-2.06) | 2.6  (1.0-4.2) | -0.4  (-2.0-1.3) | 0.87  (0.33-1.42) | 4.4  (2.7-6.1) | 0.8  (-0.9-2.6) | 1.23  (0.73-1.72) |
| Pain- 0 | 4.9  (3.7-6.1) | 3.1  (1.8-4.4) | 2.74  (1.81-3.67) | 7.9  (6.9-8.9) | 5.8  (4.7-6.8) | 3.67  (2.93-4.40) | 8.0  (7.2-8.7) | 5.0  (4.2-5.8) | 2.68  (2.28-3.09) | 9.8  (9.1-10.5) | 6.2  (5.5-7.0) | 2.75  (2.42-3.09) |
| Pain- 1 | 5.0  (2.8-7.3) | 3.2  (0.9-5.5) | 2.80  (1.38-4.23) | 10.3  (7.4-13.2) | 8.1  (5.3-11.0) | 4.77  (3.25-6.29) | 13.2  (10.8-15.6) | 10.2  (7.8-12.6) | 4.46  (3.49-5.40) | 12.4  (10.7-14.0) | 8.8  (7.1-10.5) | 3.47  (2.90-4.05) |
| Pain- ≥2 | 5.9  (1.9-10.0) | 4.2  (0.1-8.2) | 3.32  (0.95-5.69) | 12.7  (9.0-16.4) | 10.5  (6.9-14.2) | 5.88  (3.94-7.83) | 14.9  (11.9-18.0) | 12.0  (8.9-15.0) | 5.04  (3.86-6.21) | 15.7  (13.6-17.9) | 12.2  (10.0-14.3) | 4.42  (3.67-5.17) |
| **Women** |  |  |  |  |  |  |  |  |  |  |  |  |
| Insomnia symptoms^b^ |  |  |  |  |  |  |  |  |  |  |  |  |
| No Pain- 0 | 1.7  (1.4-2.1) | Ref. | Ref. | 3.1  (2.8-3.5) | Ref. | Ref. | 4.2  (3.8-4.7) | Ref. | Ref. | 5.2  (4.7-5.7) | Ref. | Ref. |
| No Pain- 1 | 2.0  (1.1-2.8) | 0.2  (-0.7-1.1) | 1.14  (0.61-1.66) | 2.3  (1.8-3.3) | -0.6  (-1.4-0.3) | 0.82  (0.56-1.08) | 4.6  (3.5-5.8) | 0.4  (-0.8-1.6) | 1.10  (0.81-1.39) | 6.3  (5.1-7.5) | 1.1  (-0.2-2.4) | 1.21  (0.96-1.46) |
| No Pain- ≥2 | 2.3  (1.0-3.6) | 0.5  (-0.8-1.9) | 1.31  (0.53-2.08) | 5.3  (3.3-7.3) | 2.2  (0.2-4.2) | 1.69  (1.03-2.36) | 6.2  (4.3-8.0) | 1.9  (0.0-3.8) | 1.46  (0.99-1.92) | 6.9  (5.2-7.5) | 1.7  (-0.1-3.5) | 1.32  (0.97-1.68) |
| Pain- 0 | 5.7  (4.7-6.8) | 4.0  (2.9-5.1) | 3.30  (2.48-4.13) | 10.7  (9.7-11.7) | 7.6  (6.5-8.6) | 3.42  (2.90-3.94) | 11.5  (10.7-12.4) | 7.3  (6.4-8.2) | 2.74  (2.40-3.07) | 14.3  (13.5-15.0) | 9.1  (8.1-10.0) | 2.74  (2.45-3.03) |
| Pain- 1 | 6.8  (4.9-8.8) | 5.1  (3.1-7.1) | 3.93  (2.59-5.27) | 11.9  (9.9-13.9) | 8.7  (6.7-10.8) | 3.79  (3.00-4.59) | 14.6  (12.8-16.3) | 10.3  (8.5-12.2) | 3.45  (2.90-4.00) | 16.6  (15.2-18.0) | 11.4  (9.9-12.9) | 3.19  (2.80-3.58) |
| Pain- ≥2 | 9.4  (6.6-12.3) | 7.7  (3.5-6.9) | 5.43  (2.48-7.37) | 15.0  (12.1-17.9) | 11.9  (8.9-14.8) | 4.79  (3.70-5.88) | 19.4  (17.1-21.7) | 15.2  (12.9-17.5) | 4.60  (3.89-5.32) | 17.4  (16.0-18.9) | 12.2  (10.7-13.7) | 3.35  (2.94-3.76) |

Abbreviations: CI= confidence interval; RD= risk difference; RR= relative risk; Ref.= reference.
^a^ Reported chronic MSK pain lasting for at least 3 months during the past 12 months.
^b^ Reported at least several times a week.

**Table S4.** Annual proportions (risks), risk differences (RDs) and relative risks (RRs) of long-term sickness absence due to musculoskeletal (MSK) disorders according to chronic MSK pain^a^ in combination with physical activity or insomnia symptoms in different age groups, by sex. 2 years of follow up.

|  | <30 years | | | 30-39 years | | | 40-49 years | | | ≥50 years | | |
| --- | --- | --- | --- | --- | --- | --- | --- | --- | --- | --- | --- | --- |
|  | Risk  (95% CI) | RD  (95% CI) | RR  (95% CI) | Risk  (95% CI) | RD  (95% CI) | RR  (95% CI) | Risk  (95% CI) | RD  (95% CI) | RR  (95% CI) | Risk  (95% CI) | RD  (95% CI) | RR  (95% CI) |
| **Men** |  |  |  |  |  |  |  |  |  |  |  |  |
| Physical activity^b^ |  |  |  |  |  |  |  |  |  |  |  |  |
| No Pain- High | 1.8  (1.2-2.4) | Ref. | Ref. | 1.5  (1.0-1.9) | Ref. | Ref. | 1.8  (1.3-2.2) | Ref. | Ref. | 2.8  (2.3-3.4) | Ref. | Ref. |
| No Pain- Moderate | 1.4  (0.3-2.4) | -0.4  (-1.7-0.8) | 0.76  (0.12-1.39) | 2.0  (1.0-2.9) | 5.0  (-0.6-1.6) | 1.35  (0.53-2.16) | 2.8  (1.9-3.8) | 1.1  (0.0-2.2) | 1.61  (0.93-2.30) | 2.3  (1.5-3.2) | -0.5  (-1.5-0.5) | 0.82  (0.48-1.15) |
| No Pain- Inactive/low | 1.1  (0.5-1.8) | -0.7  (-1.6-0.2) | 0.63  (0.21-1.05) | 1.8  (1.2-2.4) | 0.4  (-0.4-1.1) | 1.26  (0.68-1.85) | 3.3  (2.6-4.1) | 1.6  (0.7-2.5) | 1.90  (1.24-2.56) | 3.1  (2.4-3.7) | 0.2  (-0.6-1.1) | 1.08  (0.77-1.40) |
| Pain- High | 5.3  (3.6-7.0) | 3.5  (1.7-5.3) | 2.94  (1.56-4.33) | 7.8  (6.2-9.4) | 6.3  (4.6-8.0) | 5.38  (3.29-7.47) | 9.4  (8.0-10.8) | 7.6  (6.2-9.1) | 5.35  (3.76-6.93) | 10.7  (9.5-11.9) | 7.8  (6.5-9.2) | 3.76  (2.93-4.59) |
| Pain- Moderate | 6.1  (2.3-9.8) | 4.3  (0.5-8.0) | 3.37  (1.00-5.73) | 12.4  (8.8-16.0) | 10.9  (7.3-14.6) | 8.56  (4.79-12.33) | 10.7  (8.3-13.2) | 9.0  (6.5-11.4) | 6.11  (4.03-8.20) | 10.2  (8.4-12.1) | 7.4  (5.4-9.3) | 3.59  (2.65-4.54) |
| Pain- Inactive/low | 5.1  (3.2-6.9) | 3.2  (1.3-5.2) | 2.80  (1.38-4.23) | 9.9  (8.0-11.8) | 8.4  (6.5-10.4) | 6.82  (4.23-9.42) | 10.0  (8.6-11.3) | 8.2  (6.8-9.6) | 5.68  (4.04-7.33) | 13.3  (12.1-14.5) | 10.4  (9.1-11.8) | 4.68  (3.69-5.66) |
| Insomnia symptoms^c^ |  |  |  |  |  |  |  |  |  |  |  |  |
| No Pain- No | 1.6  (1.1-2.1) | Ref. | Ref. | 1.6  (1.3-2.0) | Ref. | Ref. | 2.4  (2.0-2.9) | Ref. | Ref. | 2.7  (2.3-3.1) | Ref. | Ref. |
| No Pain- Yes | 1.1  (0.2-1.9) | -0.6  (-1.5-0.4) | 0.66  (0.11-1.20) | 2.0  (0.9-3.0) | 0.3  (-0.8-1.4) | 1.19  (0.51-1.87) | 2.8  (1.7-4.0) | 0.4  (-0.9-1.6) | 1.15  (0.64-1.67) | 3.4  (2.4-4.4) | 0.7  (-0.5-1.8) | 1.24  (0.82-1.66) |
| Pain- No | 5.2  (3.8-6.5) | 3.6  (2.1-5.0) | 3.23  (1.94-5.52) | 8.4  (7.1-9.7) | 6.7  (5.4-8.1) | 5.11  (3.69-6.52) | 8.6  (7.6-9.5) | 6.1  (5.1-7.2) | 3.51  (2.80-4.22) | 10.3  (9.5-11.2) | 7.6  (6.6-8.5) | 3.77  (3.13-4.42) |
| Pain- Yes | 5.8  (3.3-8.3) | 4.2  (1.6-6.7) | 3.61  (1.71-5.50) | 12.2  (9.5-15.0) | 10.6  (7.8-13.4) | 7.45  (5.06-9.84) | 13.9  (11.7-16.0) | 11.4  (9.2-13.6) | 5.67  (4.36-6.99) | 15.2  (13.6-16.8) | 12.5  (10.8-14.1) | 5.55  (4.54-6.57) |
| **Women** |  |  |  |  |  |  |  |  |  |  |  |  |
| Physical activity^b^ |  |  |  |  |  |  |  |  |  |  |  |  |
| No Pain- High | 1.3  (0.9-1.7) | Ref. | Ref. | 2.9  (2.3-3.5) | Ref. | Ref. | 3.5  (2.9-4.1) | Ref. | Ref. | 3.7  (3.2-4.3) | Ref. | Ref. |
| No Pain- Moderate | 1.0  (0.3-1.7) | -0.3  (-1.1-0.5) | 0.77  (0.21-1.34) | 1.9  (1.0-2.7) | -1.1  (-2.1- -0.0) | 0.64  (0.32-0.96) | 2.9  (1.9-3.9) | -0.6  (-1.8-0.6) | 0.82  (0.50-1.14) | 2.6  (1.6-3.7) | -1.1  (-2.3- -0.1) | 0.70  (0.41-1.00) |
| No Pain- Inactive/low | 1.7  (1.1-2.4) | 4.0  (-0.4-1.2) | 1.30  (0.66-1.93) | 2.8  (2.1-3.5) | -0.1  (-1.1-0.8) | 0.95  (0.65-1.26) | 3.7  (2.9-4.5) | 0.2  (-0.9-1.2) | 1.04  (0.75-1.34) | 4.6  (3.7-5.4) | 0.8  (-0.2-1.9) | 1.22  (0.92-1.53) |
| Pain- High | 6.0  (4.7-7.4) | 4.7  (3.3-6.1) | 4.50  (2.83-6.17) | 13.5  (11.9-15.1) | 10.6  (8.9-12.3) | 4.65  (3.57-5.73) | 13.1  (11.2-14.3) | 9.6  (8.2-10.9) | 3.70  (2.99-4.42) | 15.1  (14.1-16.2) | 11.4  (10.2-12.6) | 4.04  (3.34-4.75) |
| Pain- Moderate | 4.5  (2.5-6.4) | 3.1  (1.2-5.1) | 3.33  (1.59-5.06) | 11.9  (9.3-14.5) | 9.0  (6.3-11.7) | 4.10  (2.88-5.32) | 13.2  (11.0-15.4) | 9.7  (7.4-11.9) | 3.74  (2.85-4.62) | 13.7  (11.8-15.5) | 9.9  (8.0-11.8) | 3.65  (2.88-4.41) |
| Pain- Inactive/low | 6.7  (4.9-8.4) | 5.3  (3.5-7.1) | 4.98  (3.00-6.96) | 11.4  (9.8-13.0) | 8.5  (6.8-10.2) | 3.93  (2.96-4.89) | 15.9  (14.4-17.4) | 12.4  (10.8-14.0) | 4.50  (3.62-5.38) | 17.3  (15.7-18.3) | 13.3  (11.9-14.7) | 4.55  (3.74-5.36) |
| Insomnia symptoms^c^ |  |  |  |  |  |  |  |  |  |  |  |  |
| No Pain- No | 1.7  (1.0-1.6) | Ref. | Ref. | 2.5  (2.1-2.9) | Ref. | Ref. | 3.4  (2.9-3.8) | Ref. | Ref. | 3.8  (3.3-4.3) | Ref. | Ref. |
| No Pain- Yes | 1.8  (1.0-2.6) | 0.5  (-0.4-1.4) | 1.38  (0.68-2.08) | 3.4  (2.3-4.5) | 0.9  (-0.3-2.0) | 1.35  (0.87-1.83) | 4.1  (3.0-5.2) | 0.7  (-0.5-1.9) | 1.21  (0.84-1.58) | 4.0  (3.1-5.0) | 0.2  (-0.9-1.3) | 1.05  (0.76-1.34) |
| Pain- No | 5.4  (4.3-6.5) | 4.1  (2.9-5.2) | 4.13  (2.78-5.47) | 11.2  (10.0-12.5) | 8.7  (7.5-10.0) | 4.48  (3.57-5.39) | 12.2  (11.2-13.2) | 8.8  (7.7-9.9) | 3.62  (3.03-4.22) | 14.6  (13.7-15.6) | 10.8  (9.7-11.9) | 3.84  (3.26-4.42) |
| Pain- Yes | 7.0  (5.2-8.7) | 5.7  (3.9-7.5) | 5.36  (3.42-7.29) | 15.0  (12.9-17.0) | 12.5  (10.4-14.5) | 5.96  (4.65-7.27) | 17.8  (16.1-19.4) | 14.4  (12.7-16.1) | 5.28  (4.38-6.18) | 16.9  (15.7-18.1) | 13.1  (11.8-14.4) | 4.43  (3.76-5.11) |

Abbreviations: CI= confidence interval; RD= risk difference; RR= relative risk; Ref.= reference.
^a^ Reported chronic MSK pain lasting for at least 3 months during the past 12 months.
^b^ “inactive/low” (<3 hours light and no hard activity), “moderate” (at least ≥3 hours light and/or <1 hour hard activity), and “high” (any light and ≥1 hour hard activity)
^c^ Reported at least one insomnia symptom several times a week.

**Table S5.** Annual proportions (risks), risk differences (RDs) and relative risks (RRs) of long-term sickness absence including disability benefits due to musculoskeletal (MSK) disorders according to chronic MSK pain^a^ in combination with physical activity or insomnia symptoms in different age groups, by sex.

|  | <30 years | | | 30-39 years | | | 40-49 years | | | ≥50 years | | |
| --- | --- | --- | --- | --- | --- | --- | --- | --- | --- | --- | --- | --- |
|  | Risk  (95% CI) | RD  (95% CI) | RR  (95% CI) | Risk  (95% CI) | RD  (95% CI) | RR  (95% CI) | Risk  (95% CI) | RD  (95% CI) | RR  (95% CI) | Risk  (95% CI) | RD  (95% CI) | RR  (95% CI) |
| **Men** |  |  |  |  |  |  |  |  |  |  |  |  |
| Physical activity^b^ |  |  |  |  |  |  |  |  |  |  |  |  |
| No Pain- High | 1.9  (1.4-2.5) | Ref. | Ref. | 2.2  (1.8-2.6) | Ref. | Ref. | 2.5  (2.1-2.9) | Ref. | Ref. | 3.7  (3.3-4.2) | Ref. | Ref. |
| No Pain- Moderate | 1.8  (0.8-2.8) | -0.2  (-1.3-0.9) | 0.91  (0.35-1.48) | 2.5  (1.6-3.4) | 0.3  (-0.7-1.3) | 1.15  (0.69-1.61) | 3.3  (2.5-4.0) | 0.7  (-0.2-1.6) | 1.28  (0.92-1.65) | 4.1  (3.3-5.0) | 0.4  (-0.6-1.4) | 1.11  (0.83-1.38) |
| No Pain- Inactive/low | 1.5  (0.9-2.2) | -0.4  (-1.2-0.4) | 0.79  (0.40-1.18) | 2.5  (1.9-3.0) | 0.3  (-0.4-1.0) | 1.13  (0.80-1.46) | 4.2  (3.6-4.8) | 1.7  (0.9-2.4) | 1.66  (1.30-2.01) | 4.4  (3.8-5.1) | 0.7  (-0.1-1.5) | 1.19  (0.97-1.42) |
| Pain- High | 4.9  (3.5-6.4) | 3.0  (1.4-4.6) | 2.55  (1.50-3.59) | 7.6  (6.2-9.0) | 5.4  (4.0-6.9) | 3.48  (2.53-4.43) | 9.3  (8.1-10.5) | 6.8  (5.5-8.0) | 3.68  (2.94-4.41) | 12.3  (11.2-13.4) | 8.6  (7.4-9.8) | 3.29  (2.78-3.80) |
| Pain- Moderate | 5.9  (2.4-9.4) | 4.0  (0.5-7.5) | 3.05  (1.06-5.03) | 9.6  (7.8-12.2) | 7.5  (4.8-10.1) | 4.41  (2.93-5.89) | 11.2  (9.0-13.4) | 8.7  (6.5-10.9) | 4.42  (3.32-5.52) | 14.0  (12.1-15.9) | 10.3  (8.3-12.2) | 3.75  (3.05-4.45) |
| Pain- Inactive/low | 5.0  (3.4-6.6) | 3.0  (1.4-4.7) | 2.56  (1.46-3.66) | 8.8  (7.3-10.4) | 6.7  (5.1-8.2) | 4.05  (2.97-5.12) | 10.4  (9.2-11.6) | 7.9  (6.6-9.1) | 4.11  (3.32-4.90) | 16.6  (15.4-17.8) | 12.9  (11.6-14.2) | 4.45  (3.79-5.11) |
| Insomnia symptoms^c^ |  |  |  |  |  |  |  |  |  |  |  |  |
| No Pain- No | 1.8  (1.4-2.2) | Ref. | Ref. | 2.2  (1.9-2.6) | Ref. | Ref. | 3.2  (2.8-3.5) | Ref. | Ref. | 3.9  (3.5-4.3) | Ref. | Ref. |
| No Pain- Yes | 1.8  (0.8-2.7) | -0.0  (-1.0-1.0) | 0.99  (0.42-1.56) | 3.0  (2.0-3.9) | 0.7  (-0.3-1.8) | 1.32  (0.84-1.81) | 3.6  (2.6-4.6) | 0.4  (-0.6-1.5) | 1.13  (0.80-1.47) | 4.7  (3.7-5.7) | 0.8  (-0.3-1.8) | 1.20  (0.93-1.48) |
| Pain- No | 5.0  (3.8-6.3) | 3.3  (2.0-4.6) | 2.83  (1.86-3.79) | 7.8  (6.8-8.8) | 5.6  (4.5-6.7) | 3.49  (2.79-4.19) | 8.5  (7.7-9.3) | 5.4  (4.5-6.2) | 2.68  (2.30-3.07) | 12.2  (11.4-13.1) | 8.3  (7.4-9.2) | 3.14  (2.77-3.51) |
| Pain- Yes | 5.2  (3.3-7.2) | 3.4  (1.4-5.4) | 2.93  (1.63-4.23) | 10.8  (8.5-13.0) | 8.5  (6.2-10.8) | 4.81  (3.56-6.07) | 15.1  (13.2-17.1) | 12.0  (10.0-14.0) | 4.76  (3.95-5.57) | 19.8  (18.1-21.5) | 15.9  (14.2-17.6) | 5.07  (4.42-5.73) |
| **Women** |  |  |  |  |  |  |  |  |  |  |  |  |
| Physical activity^b^ |  |  |  |  |  |  |  |  |  |  |  |  |
| No Pain- High | 1.8  (1.4-2.1) | Ref. | Ref. | 3.4  (2.9-3.9) | Ref. | Ref. | 4.9  (4.4-5.5) | Ref. | Ref. | 6.4  (5.8-7.0) | Ref. | Ref. |
| No Pain- Moderate | 2.1  (1.2-2.9) | 0.3  (-0.6-1.3) | 1.19  (0.64-1.74) | 3.4  (2.5-4.4) | 0.0  (-1.1-1.1) | 1.00  (0.68-1.33) | 4.9  (3.9-5.9) | 0.0  (-1.1-1.2) | 1.00  (0.77-1.23) | 5.0  (3.9-6.1) | -1.4  (-2.7- -0.2) | 0.78  (0.59-0.97) |
| No Pain- Inactive/low | 2.1  (1.5-2.7) | 0.3  (-0.4-1.1) | 1.19  (0.75-1.63) | 3.6  (3.0-4.3) | 0.2  (-0.6-1.0) | 1.07  (0.82-1.31) | 5.6  (4.8-6.4) | 0.7  (-0.3-1.7) | 1.14  (0.93-1.35) | 7.9  (6.9-8.8) | 1.5  (0.4-2.6) | 1.23  (1.04-1.42) |
| Pain- High | 6.3  (5.1-7.5) | 4.6  (3.3-5.8) | 3.60  (2.57-4.63) | 11.9  (10.6-13.3) | 8.5  (7.1-10.0) | 3.51  (3.84-4.17) | 13.9  (12.8-14.9) | 9.0  (7.8-10.1) | 2.82  (2.44-3.21) | 20.3  (19.2-21.4) | 13.9  (12.7-15.2) | 3.18  (2.83-3.53) |
| Pain- Moderate | 6.5  (4.1-9.0) | 4.8  (2.3-7.3) | 3.74  (2.11-5.36) | 11.2  (9.0-13.4) | 7.8  (5.5-10.1) | 3.29  (2.47-4.11) | 13.9  (11.9-15.8) | 9.0  (6.9-11.0) | 2.82  (2.32-3.33) | 19.9  (18.0-21.8) | 13.5  (11.5-15.5) | 3.11  (2.69-3.54) |
| Pain- Inactive/low | 7.6  (5.8-9.4) | 5.9  (4.0-7.7) | 4.34  (2.95-5.74) | 11.6  (10.1-13.0) | 8.2  (6.6-9.7) | 3.40  (2.73-4.06) | 15.8  (14.5-17.2) | 10.9  (9.5-12.4) | 3.23  (2.77-3.68) | 24.6  (23.3-25.9) | 18.2  (16.8-19.7) | 3.85  (3.43-4.28) |
| Insomnia symptoms^c^ |  |  |  |  |  |  |  |  |  |  |  |  |
| No Pain- No | 1.8  (1.4-2.1) | Ref. | Ref. | 3.4  (3.0-3.8) | Ref. | Ref. | 5.0  (4.5-5.4) | Ref. | Ref. | 6.3  (5.8-6.8) | Ref. | Ref. |
| No Pain- Yes | 2.3  (1.6-3.1) | 0.6  (-0.3-1.4) | 1.31  (0.81-1.81) | 3.7  (2.8-4.6) | 0.4  (-0.6-1.3) | 1.11  (0.81-1.40) | 5.8  (4.7-6.9) | 0.8  (-0.03-2.0) | 1.17  (0.93-1.41) | 7.8  (6.7-8.9) | 1.5  (0.3-2.7) | 1.24  (1.03-1.44) |
| Pain- No | 6.0  (4.9-7.1) | 4.2  (3.1-5.3) | 3.39  (2.48-4.26) | 10.8  (9.8-11.8) | 7.4  (6.3-8.5) | 3.19  (2.70-3.68) | 12.5  (11.7-13.3) | 7.6  (6.6-8.5) | 2.53  (2.24-2.81) | 19.4  (18.5-20.4) | 13.1  (12.0-14.2) | 3.08  (2.78-3.38) |
| Pain- Yes | 8.2  (6.5-9.9) | 6.4  (4.6-8.2) | 4.63  (3.31-5.95) | 13.7  (11.9-15.4) | 10.3  (8.4-12.1) | 4.04  (3.32-4.75) | 18.7  (17.2-20.3) | 13.8  (12.2-15.4) | 3.79  (3.32-4.26) | 25.1  (23.8-26.3) | 18.8  (17.4-20.1) | 3.98  (3.58-4.37) |

Abbreviations: CI= confidence interval; RD= risk difference; RR= relative risk; Ref.= reference.
^a^ Reported chronic MSK pain lasting for at least 3 months during the past 12 months.
^b^ “inactive/low” (<3 hours light and no hard activity), “moderate” (at least ≥3 hours light and/or <1 hour hard activity), and “high” (any light and ≥1 hour hard activity).
^c^ Reported at least one insomnia symptom several times a week.

**Table S6.** Annual proportions (risks), risk differences (RDs) and relative risks (RRs) of long-term sickness absence due to all-cause according to chronic musculoskeletal pain^a^ in combination with physical activity or insomnia symptoms in different age groups, by sex.

|  | <30 years | | | 30-39 years | | | 40-49 years | | | ≥50 years | | |
| --- | --- | --- | --- | --- | --- | --- | --- | --- | --- | --- | --- | --- |
|  | Risk  (95% CI) | RD  (95% CI) | RR  (95% CI) | Risk  (95% CI) | RD  (95% CI) | RR  (95% CI) | Risk  (95% CI) | RD  (95% CI) | RR  (95% CI) | Risk  (95% CI) | RD  (95% CI) | RR  (95% CI) |
| **Men** |  |  |  |  |  |  |  |  |  |  |  |  |
| Physical activity^b^ |  |  |  |  |  |  |  |  |  |  |  |  |
| No Pain- High | 5.4  (4.5-6.3) | Ref. | Ref. | 7.0  (6.1-7.9) | Ref. | Ref. | 7.4  (6.7-8.1) | Ref. | Ref. | 9.6  (8.9-10.4) | Ref. | Ref. |
| No Pain- Moderate | 4.8  (2.9-6.6) | -0.7  (-2.7-1.4) | 0.88  (0.50-1.25) | 7.0  (5.5-8.6) | 0.0  (-1.8-1.8) | 1.00  (0.75-1.26) | 8.8  (7.4-10.2) | 1.4  (-0.2-3.0) | 1.19  (0.97-1.42) | 11.2  (9.7-12.7) | 1.6  (-0.1-3.2) | 1.16  (0.99-1.34) |
| No Pain- Inactive/low | 6.0  (4.6-7.5) | 0.6  (-1.1-2.4) | 1.11  (0.78-1.44) | 7.2  (6.3-8.2) | 0.3  (-1.0-1.5) | 1.04  (0.85-1.22) | 9.2  (8.3-10.1) | 1.8  (0.6-3.0) | 1.25  (1.07-1.42) | 10.4  (9.5-11.3) | 0.8  (-0.4-1.9) | 1.08  (0.95-1.21) |
| Pain- High | 9.7  (7.6-11.7) | 4.3  (2.0-6.5) | 1.79  (1.31-2.27) | 14.9  (13.0-16.8) | 7.9  (5.8-10.0) | 2.13  (1.75-2.51) | 15.2  (13.8-16.6) | 7.9  (6.3-9.4) | 2.07  (1.79-2.34) | 17.5  (16.3-18.6) | 7.8  (6.5-9.2) | 1.81  (1.63-2.00) |
| Pain- Moderate | 8.4  (4.6-12.3) | 3.0  (-0.9-7.0) | 1.56  (0.80-2.32) | 15.0 (12.0-18.0) | 8.0  (4.8-11.1) | 2.14  (1.63-2.65) | 18.2  (15.6-20.8) | 10.8  (8.1-13.5) | 2.46  (2.03-2.89) | 19.3  (17.4-21.2) | 9.7  (7.6-11.7) | 2.01  (1.75-2.26) |
| Pain- Inactive/low | 15.2  (12.2-18.2) | 9.8  (6.7-12.9) | 2.81  (2.09-3.54) | 16.1  (14.2-18.0) | 9.1  (7.0-11.2) | 2.30  (1.90-2.70) | 16.0  (14.6-17.4) | 8.6  (7.1-10.2) | 2.17  (1.89-2.45) | 21.1  (19.9-22.3) | 11.5  (10.0-12.9) | 2.19  (1.98-2.40) |
| Insomnia symptoms^c^ |  |  |  |  |  |  |  |  |  |  |  |  |
| No Pain- No | 4.6  (3.9-5.3) | Ref. | Ref. | 6.0  (5.5-6.6) | Ref. | Ref. | 7.6  (7.0-8.2) | Ref. | Ref. | 9.3  (8.8-9.9) | Ref. | Ref. |
| No Pain- Yes | 11.4  (8.3-14.4) | 6.8  (3.6-9.9) | 2.46  (1.71-3.22) | 14.1  (11.6-16.7) | 8.1  (5.5-10.7) | 2.34  (1.87-2.82) | 12.2  (10.4-14.1) | 4.6  (2.7-6.6) | 1.61  (1.33-1.88) | 14.0  (12.4-15.6) | 4.7  (3.0-6.4) | 1.50  (1.30-1.69) |
| Pain- No | 9.2  (7.5-10.8) | 4.6  (2.8-6.4) | 1.99  (1.52-2.45) | 13.5  (12.2-14.8) | 7.5  (6.1-8.9) | 2.24  (1.94-2.55) | 13.9  (12.9-14.9) | 6.3  (5.2-7.4) | 1.83  (1.65-2.02) | 17.2  (16.4-18.0) | 7.9  (6.8-8.9) | 1.84  (1.70-1.99) |
| Pain- Yes | 18.0  (14.4-21.6) | 13.4  (9.7-17.0) | 3.90  (2.93-4.86) | 21.4  (18.5-24.4) | 15.4  (12.4-18.4) | 3.56  (2.97-4.16) | 22.7  (20.5-25.0) | 15.1  (12.8-17.5) | 2.99  (2.62-3.36) | 24.6  (23.0-26.2) | 15.0  (13.5-16.9) | 2.63  (2.40-2.87) |
| **Women** |  |  |  |  |  |  |  |  |  |  |  |  |
| Physical activity^b^ |  |  |  |  |  |  |  |  |  |  |  |  |
| No Pain- High | 10.2  (9.3-11.1) | Ref. | Ref. | 15.5  (14.5-16.5) | Ref. | Ref. | 12.6  (11.8-13.5) | Ref. | Ref. | 15.3  (14.4-16.2) | Ref. | Ref. |
| No Pain- Moderate | 12.7  (10.7-14.8) | 2.6  (0.3-4.8) | 1.25  (1.02-1.48) | 17.9  (15.9-19.8) | 2.4  (0.3-4.6) | 1.16  (1.01-1.30) | 13.5  (11.7-15.2) | 0.8  (-1.1-2.8) | 1.07  (0.91-1.22) | 14.9  (13.1-16.7) | -0.4  (-2.4-1.7) | 0.98  (0.85-1.11) |
| No Pain- Inactive/low | 17.0  (15.3-18.7) | 6.8  (4.9-8.7) | 1.67  (1.44-1.89) | 17.8  (16.5-19.1) | 2.3  (0.7-4.0) | 1.15  (1.04-1.26) | 14.7  (13.4-16.0) | 2.1  (0.5-3.6) | 1.17  (1.03-1.30) | 16.0  (14.8-17.2) | 0.7  (-0.9-2.2) | 1.04  (0.94-1.15) |
| Pain- High | 20.1  (18.1-22.0) | 9.9  (7.7-12.1) | 1.97  (1.71-2.23) | 27.8  (26.0-29.5) | 12.3  (10.3-14.3) | 1.80  (1.63-1.96) | 24.9  (23.6-26.2) | 12.3  (10.7-13.8) | 1.97  (1.80-2.14) | 25.7  (24.6-26.7) | 10.4  (9.0-11.8) | 1.68  (1.56-1.80) |
| Pain- Moderate | 20.3  (16.8-23.8) | 10.1  (6.5-13.7) | 1.99  (1.61-2.38) | 26.5  (23.7-29.4) | 11.1  (8.1-14.1) | 1.72  (1.50-1.93) | 25.3  (23.0-27.6) | 12.6  (10.2-15.1) | 2.00  (1.77-2.23) | 25.1  (23.3-27.0) | 9.8  (7.7-11.9) | 1.64  (1.48-1.80) |
| Pain- Inactive/low | 27.4  (24.8-30.1) | 17.3  (14.5-20.1) | 2.70  (2.34-3.05) | 29.5  (27.6-31.5) | 14.1  (11.9-16.3) | 1.91  (1.74-2.09) | 28.5  (26.9-30.1) | 15.9  (14.1-17.7) | 2.26  (2.06-2.46) | 28.3  (27.0-30.0) | 13.0  (11.4-14.6) | 1.85  (1.71-1.99) |
| Insomnia symptoms^c^ |  |  |  |  |  |  |  |  |  |  |  |  |
| No Pain- No | 11.2  (10.4-12.1) | Ref. | Ref. | 15.2  (14.4-16.0) | Ref. | Ref. | 12.4  (11.7-13.1) | Ref. | Ref. | 14.2  (13.5-15.0) | Ref. | Ref. |
| No Pain- Yes | 17.0  (15.0-19.1) | 5.8  (3.6-8.0) | 1.52  (1.31-1.73) | 22.6  (20.5-24.6) | 7.4  (5.2-9.6) | 1.49  (1.33-1.64) | 18.0  (16.1-19.9) | 5.6  (3.6-7.7) | 1.46  (1.28-1.63) | 19.5  (17.9-21.1) | 5.3  (3.5-7.1) | 1.37  (1.24-1.51) |
| Pain- No | 19.5  (17.8-21.2) | 8.2  (6.4-10.1) | 1.73  (1.54-1.93) | 25.1  (23.8-26.5) | 9.9  (8.4-11.5) | 1.65  (1.53-1.78) | 23.0  (22.0-24.0) | 10.6  (9.4-11.9) | 1.86  (1.73-1.99) | 24.3  (23.4-25.2) | 10.1  (8.9-11.3) | 1.71  (1.60-1.82) |
| Pain- Yes | 28.1  (25.4-30.8) | 16.9  (14.0-19.7) | 2.50  (2.20-2.80) | 34.9  (32.6-37.1) | 19.7  (17.3-22.1) | 2.30  (2.11-2.49) | 32.8  (31.0-34.5) | 20.4  (18.5-22.3) | 2.65  (2.44-2.86) | 29.7  (28.5-30.9) | 15.6  (14.1-16.9) | 2.09  (1.95-2.23) |

Abbreviations: CI= confidence interval; RD= risk difference; RR= relative risk; Ref.= reference.
^a^ Reported chronic MSK pain lasting for at least 3 months during the past 12 months.
^b^ “inactive/low” (<3 hours light and no hard activity), “moderate” (at least ≥3 hours light and/or <1 hour hard activity), and “high” (any light and ≥1 hour hard activity).
^c^ Reported at least one insomnia symptom several times a week.
